# Parathyroid Hormone-Related Peptide-Producing Gallbladder Cancer Presenting With Humoral Hypercalcemia of Malignancy: A Case Report

**DOI:** 10.7759/cureus.52951

**Published:** 2024-01-25

**Authors:** Koji Takahashi, Hiroshi Ohyama, Izumi Ohno, Yuichi Takiguchi, Naoya Kato

**Affiliations:** 1 Department of Gastroenterology, Chiba University, Chiba, JPN; 2 Department of Medical Oncology, Chiba University, Chiba, JPN

**Keywords:** hypercalcemia, gallbladder cancer, parathyroid hormone-related protein, humoral hypercalcemia of malignancy, cachexia

## Abstract

Humoral hypercalcemia of malignancy (HHM) is often reported in cancers derived from the squamous epithelium; however, there are very few reports of HHM in patients with gallbladder cancer. We report a case of a parathyroid hormone-related protein (PTHrP)-producing gallbladder cancer presenting with HHM. A 43-year-old woman presented with appetite loss, nausea, and brown-colored urine. Blood tests revealed that she had hypercalcemia, high serum bilirubin, and high serum parathyroid hormone. Contrast-enhanced computed tomography revealed a gallbladder tumor, liver metastasis, and bile duct obstruction caused by the gallbladder tumor in the hilar region. No bone metastasis was observed. Endoscopic retrograde cholangiopancreatography revealed pancreaticobiliary maljunction. Metal biliary stents were placed, and a transpapillary biopsy of the gallbladder tumor revealed a pathological diagnosis of adenocarcinoma. The patient was diagnosed with HHM due to gallbladder cancer with liver metastasis. Although her hypercalcemia and jaundice improved, her appetite loss and nausea did not improve. Subsequently, the patient developed disseminated intravascular coagulation, and her general condition gradually deteriorated. Due to her poor general condition, chemotherapy could not be administered. The patient died six weeks after visiting our hospital. Although rare, some gallbladder cancers cause HHM due to PTHrP production.

## Introduction

Hypercalcemia is reported to occur in 20-30% of patients with malignant tumors during the course of the disease [1]. Hypercalcemia in patients with malignant tumors can be classified into the following two types: one is humoral hypercalcemia of malignancy (HHM) caused by parathyroid hormone-related protein (PTHrP) produced from the tumor, and the other is local osteolytic hypercalcemia (LOH) caused by extensive bone destruction associated with bony tumor metastasis. As a cause of hypercalcemia associated with malignant tumors, the frequency of HHM is approximately 80% and 20%, respectively. HHM, as a paraneoplastic syndrome, is often reported in cancers derived from the squamous epithelium, such as head, neck, and lung cancers. There are few reports of HHM in patients with gallbladder cancer [2,3]. Here, we report a case of PTHrP-producing gallbladder cancer presenting with HHM.

## Case presentation

**Figure 1:**
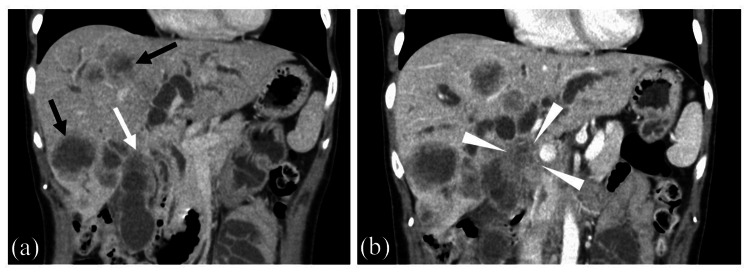
Contrast-enhanced computed tomography. (a) Contrast-enhanced computed tomography revealed a gallbladder tumor (white arrow) and liver metastases (black arrow). (b) Bile ducts in the hilar region were obstructed by a gallbladder tumor (arrowhead).

A 43-year-old woman developed loss of appetite, nausea, epigastric discomfort, and brown-colored urine and visited a local hospital near her home. The patient's medical history was unremarkable, and she was not taking any oral medications, including supplements or herbal medicines. She worked in an office, and she was not exposed to chemicals. Blood tests revealed that she had hypercalcemia and high serum bilirubin (Table 1). Contrast-enhanced computed tomography (CE-CT) revealed a gallbladder tumor, liver metastases, and bile duct obstruction caused by a gallbladder tumor in the hilar region (Figure 1).

**Table 1:** Laboratory data at the first hospital visit. WBC, white blood cells; RBC, red blood cells; Hb, hemoglobin; Hct, hematocrit; MCV, mean corpuscular volume; MCH, mean corpuscular hemoglobin; MCHC, mean corpuscular hemoglobin concentration; Plt, platelet; TP, total protein; Alb, albumin; BUN, blood urea nitrogen; Cre, creatinine; Na, sodium; K, potassium; Ca, calcium; AST, aspartate aminotransferase; ALT, alanine transaminase; LDH, lactate dehydrogenase; ALP, alkaline phosphatase; γ-GTP, gamma-glutamyl transpeptidase; T.Bil, total bilirubin; D.Bil, direct bilirubin; Amy, amylase; CK, creatine kinase; CRP, C-reactive protein.

Parameter	Value	Reference range
WBC	12,150/μL	3,500-9,700
RBC	564×10^4^/μL	376-516
Hb	12.2 g/dL	11.2-15.2
Hct	36.8%	34.3-45.2
MCV	65 fL	80-101
MCH	21.6 pg	26.4-34.3
MCHC	33.2%	31.3-36.1
Plt	33 ×10^4^/μL	14.0-37.9
TP	7.6 g/dL	6.5-8.2
Alb	4.3 g/dL	3.8-5.2
BUN	12.5 mg/dL	8-20
Cre	0.59 mg/dL	0.46-0.82
Na	134 mEq/L	135-145
K	3.2 mEq/L	3.5-5.0
Ca	15.5 mg/dL	8.6-10.2
AST	193 IU/L	10-40
ALT	433 IU/L	5-45
LDH	231 IU/L	120-245
ALP	526 IU/L	38-113
γ-GTP	479 IU/L	0-48
T.Bil	9.4 mg/dL	0.3-1.2
D.Bil	6.5 mg/dL	0-0.4
Amy	47 IU/dL	39-134
CK	37 IU/L	50-210
CRP	1.65 mg/dL	0.00-0.30

After saline infusion and bisphosphonate administration, she was referred to our hospital for evaluation and treatment of the gallbladder tumor, hypercalcemia, and obstructive jaundice. On arrival at our hospital, her symptoms showed minimal improvement. Blood tests at our hospital showed high serum PTHrP levels (20.0 pmol/L; normal range: 0-1.1 pmol/L), while the serum PTH levels were within the normal range. Although we have not performed positron emission tomography, whole-body CE-CT revealed the absence of bone metastases. We determined that the cause of hypercalcemia in this patient was HHM due to PTHrP production by the gallbladder tumor.

**Figure 2:**
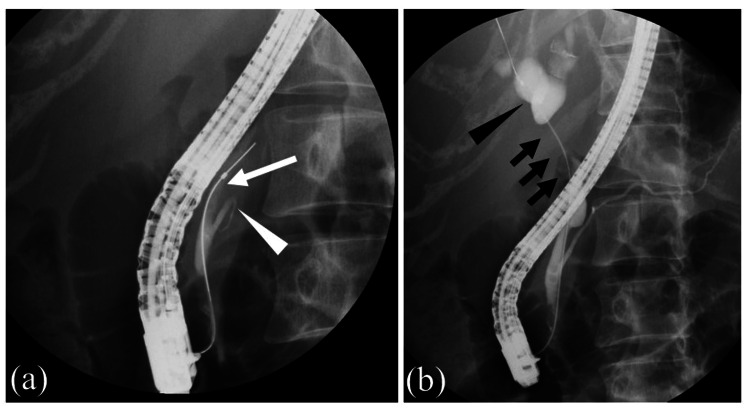
Endoscopic retrograde cholangiopancreatography. Endoscopic retrograde cholangiopancreatography was performed. (a) A catheter was inserted into the bile duct (white arrow), and cholangiography was performed. When the bile duct was imaged, the pancreatic duct (white arrowhead) was also imaged at the same time. It showed that the bile duct and pancreatic duct merged outside the duodenal wall. (b) The area of bile duct obstruction was also confirmed (black arrow). The bile duct upstream of the obstruction was dilated (black arrowhead).

**Figure 3:**
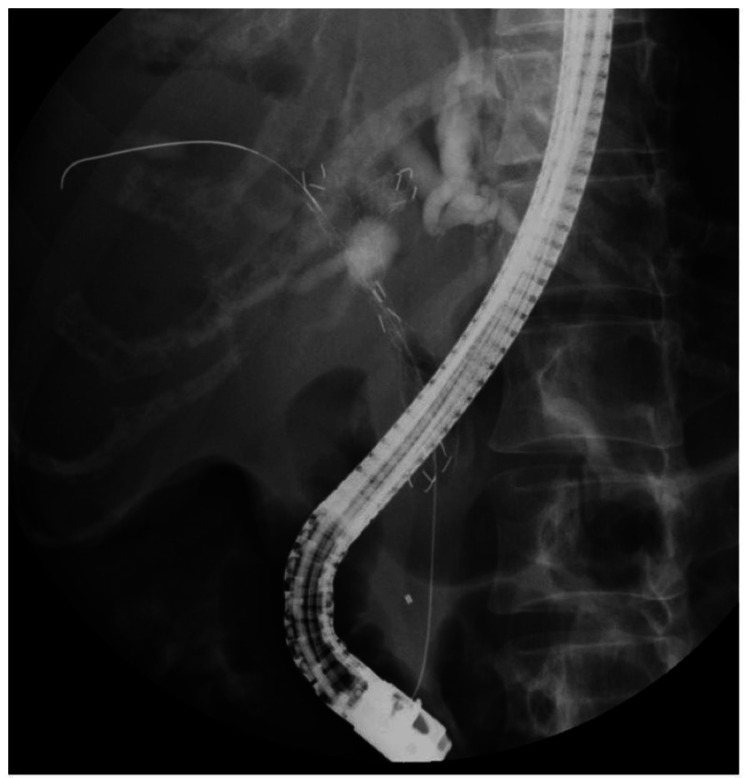
Transpapillary biliary stenting. Two metal biliary stents without covers were placed transpapillary using the side-by-side method in the left and right intrahepatic bile ducts across the hilar biliary strictures.

**Figure 4:**
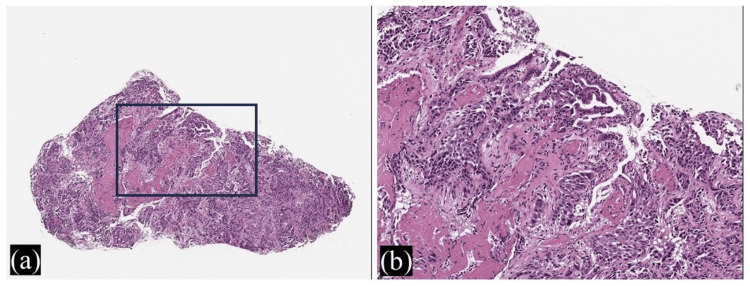
Histopathology of the gallbladder tumor obtained from biopsy during endoscopic retrograde cholangiopancreatography. Atypical cells proliferate in tubular and vesicular shapes, indicating adenocarcinoma. (a) Hematoxylin and eosin stain (40x magnification). (b) Hematoxylin and eosin stain (100x magnification).

The patient was admitted to our hospital, and an immediate infusion of physiological saline was initiated. Administration of bisphosphonates was also continued. Thereafter, hypercalcemia gradually improved. To improve obstructive jaundice, transpapillary biliary drainage using endoscopic retrograde cholangiopancreatography (ERCP) was planned. First, upper gastrointestinal endoscopy was performed using a forward-viewing endoscope. As a result, it was confirmed that there were no problems with the hypopharynx, larynx, esophagus, or stomach. Next, an oblique-viewing endoscope was inserted to perform ERCP. There was no stricture in the duodenum, and it was possible to reach the papillae of Vater using an endoscope. Cholangiography was performed after the catheter was inserted into the bile ducts. The pancreatic duct was imaged simultaneously with the bile duct. The bile and pancreatic ducts merged outside the duodenal wall, suggesting a pancreaticobiliary maljunction (PBM). An area of bile duct obstruction was confirmed (Figure 2). The bile duct in the hilar region was obstructed by a gallbladder tumor, and the left and right intrahepatic bile ducts were separated. Two metal biliary stents without covers were placed transpapillary using the side-by-side method in the left and right intrahepatic bile ducts across the hilar biliary strictures (Figure 3). Bile was collected during ERCP. Normal bile contains little amylase, but in this case, the amylase level in the bile was high at 14,294 IU/L, suggesting that pancreatic fluid was flowing into the bile duct. PBM was considered the cause of the gallbladder cancer. A transpapillary biopsy of the gallbladder tumor during ERCP revealed a pathological diagnosis of adenocarcinoma (Figure 4). The patient was diagnosed with gallbladder cancer and liver metastasis causing HHM.

Jaundice improved after biliary stenting. Although her hypercalcemia and jaundice improved, her appetite loss and nausea did not improve. Subsequently, she developed cancerous disseminated intravascular coagulation, and her general condition gradually deteriorated. Due to her poor general condition, chemotherapy could not be administered. The patient died six weeks after visiting our hospital.

## Discussion

This report describes a case of gallbladder cancer with HHM that arose from PBM. PBM is a congenital anomaly defined as the junction of the pancreatic and bile ducts located outside the duodenal wall, usually forming a very long common duct [4,5]. PBM can be divided into PBM with and without biliary dilatation based on whether the bile duct is dilated to 10 mm or more. In patients with PBM, the bile and pancreatic ducts meet immediately before reaching the duodenal wall. Therefore, the sphincter of Oddi does not function at the confluence of the bile and pancreatic ducts. In most cases, the internal pressure is higher in the pancreatic duct than in the bile duct; therefore, the pancreatic fluid flows into the bile duct. Kamisawa et al. reported that the average amylase level in the bile duct is 99,000 IU/L in patients with PBM [5]. Patients with PBM are more likely to develop biliary tract cancers due to the inflow of pancreatic fluid into the bile ducts. It was reported that gallbladder cancer was detected in 33 (67%) of 49 patients with PBM without biliary dilatation. It was also reported that the mean age at onset of gallbladder cancer in patients with PBM was 59.2 years, which was significantly younger than the mean age at onset of gallbladder cancer in patients without PBM, which was 69.4 years [5]. In this case, if PBM had been diagnosed and surgery to divide the bile duct and pancreatic duct had been performed at a young age, gallbladder cancer might not have occurred.

In this case, the diagnosis of HHM due to a PTHrP-producing tumor was made based on the absence of obvious bone metastases on imaging and high serum PTHrP levels. PTHrP was discovered through investigations of tumor-induced hypercalcemia in patients without bone metastases [6]. HHM is observed in various histological types of cancer but is particularly common in squamous cell carcinoma. It is found in approximately 10% of patients with advanced lung, head and neck, renal, breast, and bladder cancers [1,7]. PTHrP has been reported to not only increase blood calcium levels, but also promote cachexia, and there are several reports on the correlation between PTHrP and cachexia [8-11]. Kir et al. reported that PTHrP plays an important role in wasting by driving thermogenic gene expression in adipose tissues; therefore, PTHrP contributes to the broader aspects of cancer cachexia [8]. Additionally, HHM affects the prognosis of patients with cancer. The prognosis of patients with HHM is poor, with survival times estimated to be approximately one to three months from the onset of hypercalcemia [1,12]. In patients with advanced malignant tumors with hypercalcemia and high blood PTHrP levels, high blood PTHrP concentrations have been reported as a poor prognostic factor in patients aged 65 years or younger [13].

In our case, anorexia and nausea persisted throughout the clinical course. We initially thought that, in this case, if we corrected the obstructive jaundice and hypercalcemia, anorexia and nausea would improve. However, although the obstructive jaundice and hypercalcemia improved, her symptoms did not. This was determined to be due to cachexia caused by cancer. Although we cannot be certain, we believe that there is a possibility that this cachexia is affected by PTHrP caused by gallbladder cancer. Hypercalcemia causes nausea. However, if symptoms persist even after hypercalcemia improves, PTHrP-related cachexia should be considered.

## Conclusions

Herein, we report a case of PTHrP-producing gallbladder cancer presenting with HHM. Although rare, some gallbladder cancers cause HHM due to PTHrP production. PTHrP not only causes hypercalcemia but also promotes cachexia. If symptoms persist even after hypercalcemia improves, PTHrP-related cachexia should be considered.

## References

[1] Stewart AF (2005). Clinical practice. Hypercalcemia associated with cancer. N Engl J Med.

[2] Ebinuma H, Imaeda H, Fukuda Y (2002). A case of parathyroid hormone-related peptide producing gallbladder carcinoma and establishment of a cell line, PTHrP-GBK. Dig Dis Sci.

[3] Yanagi M, Suda T, Oishi N, Kobayashi M, Matsushita E (2023). Adenosquamous carcinoma of the gallbladder simultaneously producing granulocyte-colony-stimulating factor and parathyroid hormone-related protein. Clin J Gastroenterol.

[4] Kamisawa T, Egawa N, Nakajima H, Tsuruta K, Okamoto A, Matsukawa M (2005). Origin of the long common channel based on pancreatographic findings in pancreaticobiliary maljunction. Dig Liver Dis.

[5] Kamisawa T, Takuma K, Anjiki H (2009). Pancreaticobiliary maljunction. Clin Gastroenterol Hepatol.

[6] Suva LJ, Winslow GA, Wettenhall RE (1987). A parathyroid hormone-related protein implicated in malignant hypercalcemia: cloning and expression. Science.

[7] Gensure RC, Gardella TJ, Jüppner H (2005). Parathyroid hormone and parathyroid hormone-related peptide, and their receptors. Biochem Biophys Res Commun.

[8] Kir S, White JP, Kleiner S, Kazak L, Cohen P, Baracos VE, Spiegelman BM (2014). Tumour-derived PTH-related protein triggers adipose tissue browning and cancer cachexia. Nature.

[9] Iguchi H, Aramaki Y, Maruta S, Takiguchi S (2006). Effects of anti-parathyroid hormone-related protein monoclonal antibody and osteoprotegerin on PTHrP-producing tumor-induced cachexia in nude mice. J Bone Miner Metab.

[10] Iguchi H, Onuma E, Sato K, Sato K, Ogata E (2001). Involvement of parathyroid hormone-related protein in experimental cachexia induced by a human lung cancer-derived cell line established from a bone metastasis specimen. Int J Cancer.

[11] Sato K, Yamakawa Y, Shizume K (1993). Passive immunization with anti-parathyroid hormone-related protein monoclonal antibody markedly prolongs survival time of hypercalcemic nude mice bearing transplanted human PTHrP-producing tumors. J Bone Miner Res.

[12] Ralston SH, Gallacher SJ, Patel U, Campbell J, Boyle IT (1990). Cancer-associated hypercalcemia: morbidity and mortality. Clinical experience in 126 treated patients. Ann Intern Med.

[13] Truong NU, deB Edwardes MD, Papavasiliou V, Goltzman D, Kremer R (2003). Parathyroid hormone-related peptide and survival of patients with cancer and hypercalcemia. Am J Med.

